# α‐Lipoic Acid Ameliorates Arsenic‐Induced Lipid Disorders by Promoting Peroxisomal β‐Oxidation and Reducing Lipophagy in Chicken Hepatocyte

**DOI:** 10.1002/advs.202413255

**Published:** 2025-01-30

**Authors:** Yangfei Zhao, Mingyue Guo, Ting Pei, Chenqi Shang, Yirong Chen, Liying Zhao, Yiguang Lu, Chen Liang, Jundong Wang, Jianhai Zhang

**Affiliations:** ^1^ College of Veterinary Medicine Shanxi Agricultural University Taigu Shanxi 030801 China; ^2^ College of Animal Science Shanxi Agricultural University Taigu Shanxi 030801 China

**Keywords:** alpha‐lipoic acid, arsenic, hepatotoxicity, lipophagy, peroxisomal β‐oxidation

## Abstract

Liver disease poses a significant threat to global public health, with arsenic (As) recognized as a major environmental toxin contributing to liver injury. However, the specific mechanisms and the protective effects of α‐lipoic acid (LA) remain unclear. Therefore, this study employs network toxicology and network pharmacology to comprehensively analyze the hepatotoxic mechanism of As and the hepatoprotective mechanism of LA, and further verifies the mechanisms of peroxisomal β‐oxidation and lipophagy in the process. The network analysis results show that As induces liver damage mainly through autophagy, apoptosis, lipid metabolism, and oxidative stress, whereas LA exerts its hepatoprotective properties mainly by regulating lipid metabolism. Further verifications find that As inhibits SIRT1 expression, activates the P53 and Notch pathways, damages mitochondria, inhibits peroxisomal β‐oxidation, increases lipid accumulation, and enhances lipophagy in the liver, while LA intervention alleviates As‐induced lipid accumulation and enhances lipophagy by targeting SIRT1, ameliorating mitochondrial damage, enhancing peroxisomal β‐oxidation, thereby alleviating As‐induced liver damage. This study further clarifies the mechanism of As hepatotoxicity and provides a theoretical basis for LA as a potential hepatoprotective agent.

## Graphical abstract

Arsenic‐induced liver injury poses a serious threat to human health and poultry industry. This study finds that α‐lipoic acid alleviates arsenic‐induced lipid accumulation and enhances lipophagy by targeting SIRT1, ameliorating mitochondrial damage, enhancing peroxisomal β‐oxidation. This study further clarifies the mechanism of arsenic hepatotoxicity and provides a theoretical basis for α‐lipoic acid as a potential hepatoprotective agent.

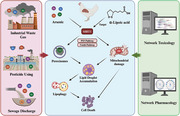

## 1 Introduction

Owing to lifestyle changes and increased environmental pollution, liver disease poses a significant threat to global public health. According to the National Institutes of Health, liver disease affects approximately 1.69 billion people globally.^[^ ^1^ ^]^ Moreover, in the poultry industry, a critical source of meat and eggs, liver disease incidence ranges from 1% to 15%, with a mortality rate between 0.5% and 10%, leading to significant economic losses.^[^ ^2^ ^]^ Arsenic (As) is a well‐known hazardous environmental metal pollutant, utilized in pesticides, herbicides, semiconductors, germicides, metal smelting, and mining.^[^ ^3^, ^4^ ^]^ As widely enters the body via water and food, accumulating through the food chain. As a key detoxification organ, the liver is a primary target for As toxicity, receiving continuous scrutiny.^[^ ^5^, ^6^ ^]^ Prolonged As exposure damages liver structure and function, disrupts metabolism, and leads to harmful substance accumulation, threatening human health and animal food safety.^[^ ^7^ ^]^ Thus, investigating the mechanisms of As‐induced liver damage and identifying effective prevention and treatment strategies are crucial for human health, poultry industry development, and animal food safety.

The liver is responsible for the de novo synthesis, oxidation, uptake, and export of lipids, providing cells with essential nutrients, and helping cells adapt to environmental changes.^[^ ^8^, ^9^ ^]^ Peroxisomes are essential organelles in hepatocytes that play a crucial role in the metabolism and detoxification processes of the liver, especially the decomposition of very long‐chain fatty acids by peroxisomal β‐oxidation.^[^ ^10^ ^]^ Peroxisomal β‐oxidation can specifically break down long‐chain fatty acids into a series of shortened acyl‐coenzyme A (CoA), generating acetyl‐CoA, propionyl‐CoA, and various medium‐chain acyl‐CoA, which are further oxidized in mitochondria to produce CO_2_ and H_2_O_2_.^[^ ^11^ ^]^ In addition, this process is regulated by the rate‐limiting enzyme ACOX1, which catalyzes the desaturation of acyl‐CoA to 2‐trans‐enoyl‐CoA, thereby generating H_2_O_2_.^[^ ^12^ ^]^ In addition, peroxisomal β‐oxidation was identified as a key factor in the pathogenesis of several liver diseases.^[^ ^13^ ^]^ For instance, ACOX1 expression is markedly reduced in the livers of patients with nonalcoholic fatty liver disease (NAFLD) and hepatocellular carcinoma, resulting in the accumulation of fatty acids in the liver, which exacerbates hepatic steatosis and malignant transformation.^[^ ^14^ ^]^ Besides, in liver cirrhosis, the decreased function of peroxisomal β‐oxidation is closely associated with hepatocyte apoptosis, fibrosis, and liver failure, and mutations or deficiencies in ACOX1 can exacerbate the pathological process of cirrhosis.^[^ ^14^ ^]^ However, the role of peroxisomal β‐oxidation in As‐induced liver damage remains unclear.

Abnormal lipid metabolism in the liver can lead to lipid droplet accumulation, which in turn induces various liver diseases. Autophagy has been found to regulate both the biogenesis and degradation of lipid droplets in lipid metabolism, a process referred to as lipophagy.^[^ ^15^ ^]^ Lipophagy is a subtype of autophagy that utilizes lysosomal acid lipase to act on lipid droplets, forming autophagosomes that sequester and subsequently fuse with lysosomes, providing the liver with the ability to convert triglycerides (TGs) and undergo fatty acid β‐oxidation, thereby preventing further lipid deposition and metabolic liver diseases.^[^ ^16^, ^17^ ^]^ For instance, studies have shown that activation of lipophagy reduces hepatic steatosis and inhibits excessive accumulation of TGs, thereby attenuating the development of NAFLD.^[^ ^18^ ^]^ Currently, the regulatory relationship between lipophagy and peroxisomal β‐oxidation in lipid metabolism has received widespread attention. For instance, studies have indicated that peroxisomes interact with the autophagy‐lysosomal system.^[^ ^19^ ^]^ Furthermore, the liver‐specific ACOX1 knockout mice model revealed that ACOX1 deficiency led to decreased acetyl‐CoA levels and acetylation of the key mTORC1 subunit Raptor, which induced autophagy through ULK1.^[^ ^20^ ^]^ However, lipophagy is a selective autophagy process that specifically removes excess lipid droplets, and there is no direct evidence to clarify the regulatory relationship between peroxisome β‐oxidation and lipophagy. Therefore, elucidating the mechanisms of lipophagy and peroxisomal β‐oxidation in As‐induced disruption of hepatic lipid metabolism is crucial for understanding As hepatotoxicity.

α‐lipoic acid (LA), commonly referred to as thioctic acid and 1,2‐dithiolane‐3‐pentanoic acid, is a naturally occurring antioxidant found in almost all types of prokaryotic and eukaryotic cells.^[^ ^21^ ^]^ It exhibits a range of biochemical functions, including antioxidant and metal‐chelating properties, and can enhance the activity of other antioxidants, such as glutathione.^[^ ^22^ ^]^ Clinically, LA is utilized in the treatment of diabetes‐related polyneuropathy, where it helps improve pain and sensory abnormalities.^[^ ^23^ ^]^ In the context of cancer prevention and therapy, LA exhibits direct antitumor activity, inducing apoptosis, inhibiting cell proliferation.^[^ ^24^ ^]^ Moreover, studies have shown that LA promotes growth, enhances antioxidant effects, regulates lipid metabolism, mitigates the effects of toxins, improves immunity, inhibits cell apoptosis, reduces endoplasmic reticulum stress, and inhibits lipid deposition both in vitro and in vivo.^[^ ^21^, ^22^ ^]^ For example, Park et al. demonstrated that LA reduced plasma and liver TG levels in rats fed a high‐fat diet.^[^ ^25^ ^]^ Furthermore, LA supplement could reduce hepatic lipid deposition, upregulate ATGL expression, and downregulate FASN and phosphorylated ACC expression, indicating its lipid‐lowering effects.^[^ ^26^ ^]^ However, the intervention effect of LA on liver damage caused by As exposure in chickens remains unclear, and its specific mechanisms are not yet fully understood.

Studies have shown that arsenic levels in chicken feed can reach up to 18 mg kg^−1^ and even higher in some poultry farms or arsenic contaminated areas, and exposure to arsenic concentrations of 0.95, 20.78, 40.67, and 60.25 mg kg^−1^ effect on chickens livers and kidneys through feed.^[^ ^27^, ^28^ ^]^ In addition, lower doses LA, such as 50 and 200 mg kg^−1^, have been shown to can improve feed intake and weight gain in chickens, while higher doses, such as 400 mg kg^−1^, enhance the chicken liver's antioxidant capacity and meat quality.^[^ ^29^ ^]^ However, excessively high doses, such as 900 mg kg^−1^, may suppress growth performance and reduce economic efficiency. So according to actual chicken production and the existing literatures, we chose 27.27 mg kg^−1^ of As and 200 and 400 mg kg^−1^ of LA to establish the experimental model.

In this study, network toxicology and network pharmacology were employed a combined approach to comprehensively elucidate the mechanisms underlying As‐induced liver injury and the protective effects of LA in this process. Furthermore, hematoxylin and eosin (HE) and Oil Red O staining, immunofluorescence double staining, flow cytometry, RT‐PCR, western blotting and other technologies were used to observe the effects of LA on As content in the liver, tissue morphology, lipid metabolism, peroxisomal β‐oxidation, and lipophagy in As‐induced liver injury. We aimed to investigate the alleviating effects of LA on As‐induced liver injury in chickens and elucidate the mechanisms involving peroxisomal β‐oxidation and lipophay in this process. This study will further clarify the underlying mechanisms of As‐induced liver injury and LA's hepatoprotective effects, providing a theoretical basis for the development of LA as a hepatoprotective drug to mitigate As‐induced liver damage.

## 2 Results

### 2.1 Network Toxicology Analysis of the Potential Mechanisms of As‐Induced Liver Damage

**Figure 1:**
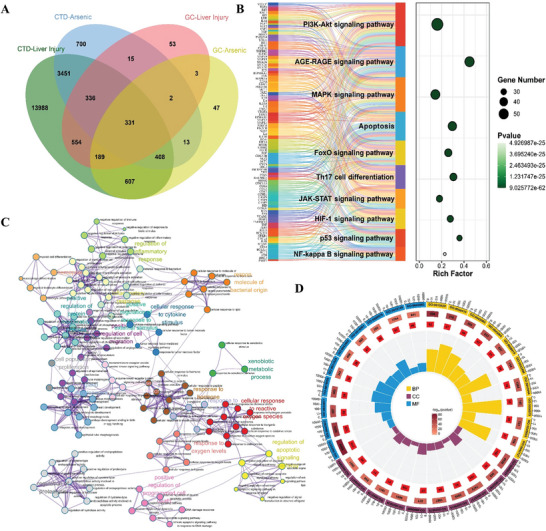
As network toxicology analysis results. A) Venn diagram for screening As‐induced liver damage related genes. B) KEGG enrichment results of As‐induced liver damage related genes. C) PPI network diagram of arsenic‐induced liver damage related genes. D) GO enrichment circle diagram of As‐induced liver damage related genes.

By utilizing the comparative toxicogenomics database (CTD) and GeneCards databases and identifying intersections, 754 As‐related target genes and 1410 liver injury‐related target genes were screened out, and a further intersection of these two datasets revealed 331 overlapping target genes (**Figure** **1**A). Gene ontology (GO) enrichment analysis revealed that, in terms of biological process (BP), the genes were primarily associated with the positive regulation of gene expression, the regulation of apoptosis, response to oxidative stress and xenobiotic stimulation, and transcriptional regulation. For cellular component (CC), the genes were localized in key subcellular regions, including the cytoplasm, nucleus, mitochondria, extracellular space, and nucleoplasm. Regarding molecular function (MF), the genes were enriched in categories such as protein binding, enzyme binding, transcription factor binding, and transferase activity (Figure 1C,D and Table S2, Supporting Information). Kyoto encyclopedia of genes and genomes (KEGG) enrichment analysis indicated involvement in key pathways, including the PI3K‐Akt signaling pathway, apoptosis process, AGE‐RAGE signaling pathway, FoxO signaling pathway, fluid shear stress and atherosclerosis, p53 signaling pathway, microRNA‐related cancer pathways, Th17 cell differentiation, HIF‐1 signaling pathway, and MAPK signaling pathway (Figure 1B and Table S3, Supporting Information). These findings suggested that As mainly affected the biological processes of hepatocytes such as oxidative stress, proliferation, differentiation, apoptosis, autophagy, lipid metabolism, and inflammation by regulating the related pathways of autophagy, apoptosis, lipid metabolism, and oxidative stress, thereby inducing liver damage.

### 2.2 LA Improved As‐Induced Liver Structural and Functional Damage

**Figure 2:**
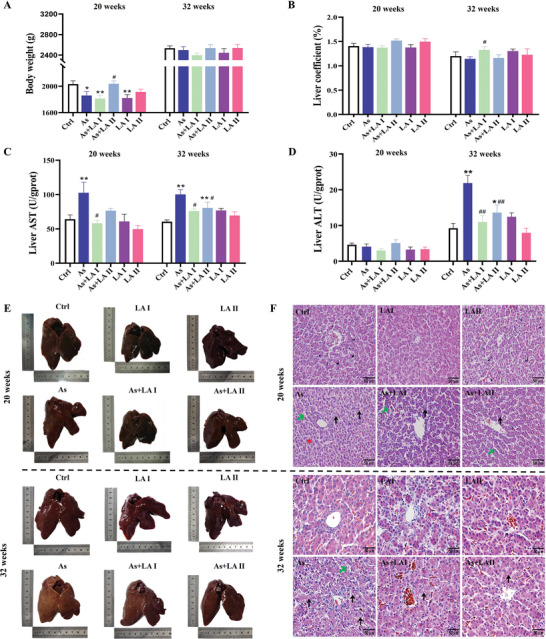
Effects of As exposure and LA intervention on growth status, liver structure and function of chickens. A) Body weight, *n* = 10. B) Liver organ coefficient, *n* = 10. C,D) Detection results of liver function index (AST and ALT), *n* = 6. E) Liver appearance observation. F) HE staining results (400×, red star: disordered hepatic cords; black arrows: lipid droplets; green arrows: enlarged intercellular spaces). ^*^ *P* < 0.05, ^**^ *P* < 0.01, versus Ctrl; ^#^ *P* < 0.05, ^##^ *P* < 0.01, versus As.

Based on network toxicology analysis and literature review, the biological functions of LA were found to overlap significantly with the hepatotoxic mechanisms of As. The results showed that after 20 weeks of treatment, As caused a decrease in chicken body weight and an increase in aspartate aminotransferase (AST) content in the liver, while the intervention of 400 mg kg^−1^ LA increased the chicken body weight and decreased the AST content in the liver compared with the As group (*P* < 0.05, *P* < 0.01, **Figure** **2**A,C). In contrast to the 20 week results, As and LA showed no significant impact on the body weight and organ coefficients after 32 weeks of treatment. Nevertheless, As obviously elevated the ASTand alanine amiotransferase (ALT) levels in the liver, and these increases were markedly alleviated by LA intervention (*P* < 0.05, *P* < 0.01, Figure 2A–D). Furthermore, pathological and morphological observations revealed that after 20 and 32 weeks of As exposure, the liver exhibited lighter coloration, disordered hepatic cord arrangement, increased lipid droplets, and elevated hepatocyte nuclear dissolution and pyknosis (Figure 2E,F). However, LA intervention effectively ameliorated the aforementioned As‐induced pathological and morphological alterations, with 400 mg kg^−1^ LA demonstrating a more pronounced protective effect (Figure 2E,F).

### 2.3 Network Pharmacology Analysis of the Potential Hepatoprotective Mechanism of LA

**Figure 3:**
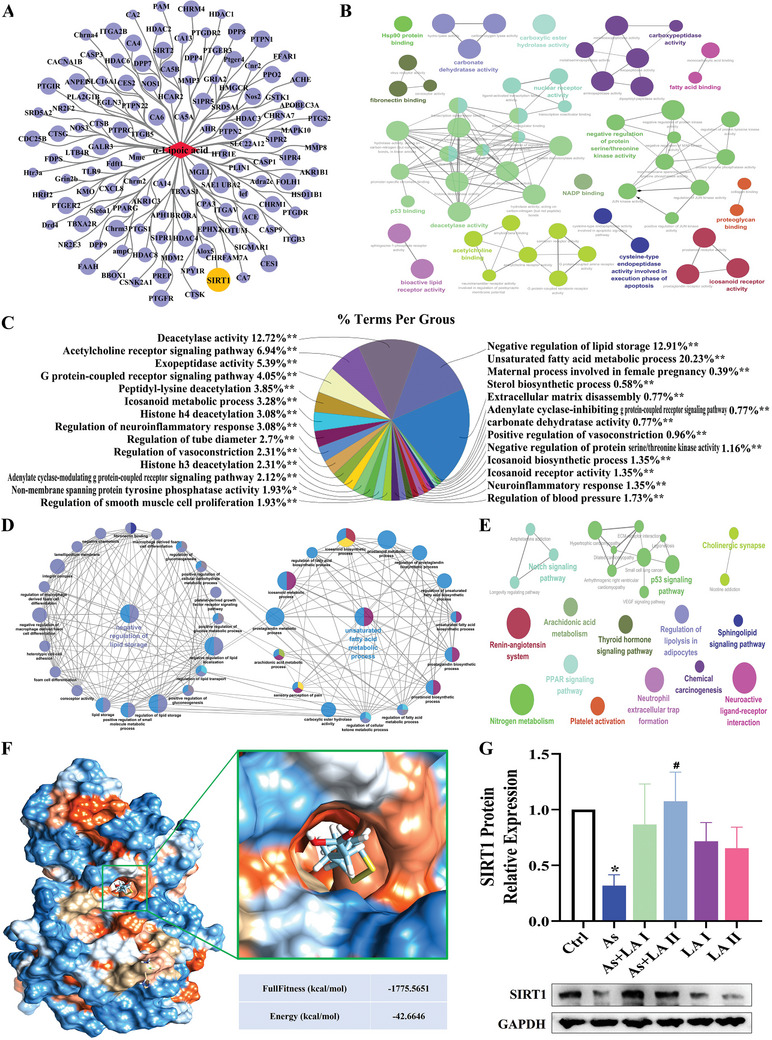
LA network pharmacology analysis results. A) Potential target genes network diagram of LA. B) GO enrichment network map of target genes. C) Analysis of the proportion of each biological process (GO enrichment). D) GO enrichment network map of lipid metabolism‐related terms. E) KEGG enrichment network map of target genes. F) Molecular docking results of LA and SIRT1. G) Detection results of SIRT1 protein expression level, *n* = 3. ^*^ *P* < 0.05, versus Ctrl; ^#^ *P* < 0.05, versus As.

To further investigate the potential hepatoprotective effects of LA, network pharmacology was employed to identify and analyze key target genes and biological characteristics associated with LA. As shown in **Figure** **3**A, 125 potential target genes with probability > 0 of LA were screened in the Swiss Target Prediction and TargetNet databases. In addition, the GO enrichment analysis results showed that LA mainly acted on terms related to lipid metabolism, among which it is most closely associated with the unsaturated fatty acid metabolic process and negative regulation of lipid storage, representing 20.23% and 19.21%, respectively (Figure 3B–D and Table S4, Supporting Information). Further, KEGG enrichment analysis of lipid metabolism‐related target genes revealed that it was mainly related to pathways such as PPAR signaling pathway, P53 signaling pathway, Notch signaling pathway, regulation of lipolysis, and sphingolipid signaling pathway (Figure 3E and Table S5, Supporting Information). In addition, analysis revealed that SIRT1 is a key target of LA, and molecular docking results indicated that the SIRT1 protein had multiple binding pockets that can stably bind to LA with low binding energy (Figure 3F,G, full fitness: ‐1775.5651 kcal mol^−1^, energy: ‐42.6646 kcal mol^−1^). Protein validation showed that the expression level of SIRT1 protein was significantly decreased after As exposure, but this change was reversed by LA intervention. These results indicated that LA primarily ameliorates liver damage by modulating lipid metabolism and SIRT1 plays a vital role in this process.

### 2.4 LA Improved As‐Induced Liver Lipid Metabolism Disorder

**Figure 4:**
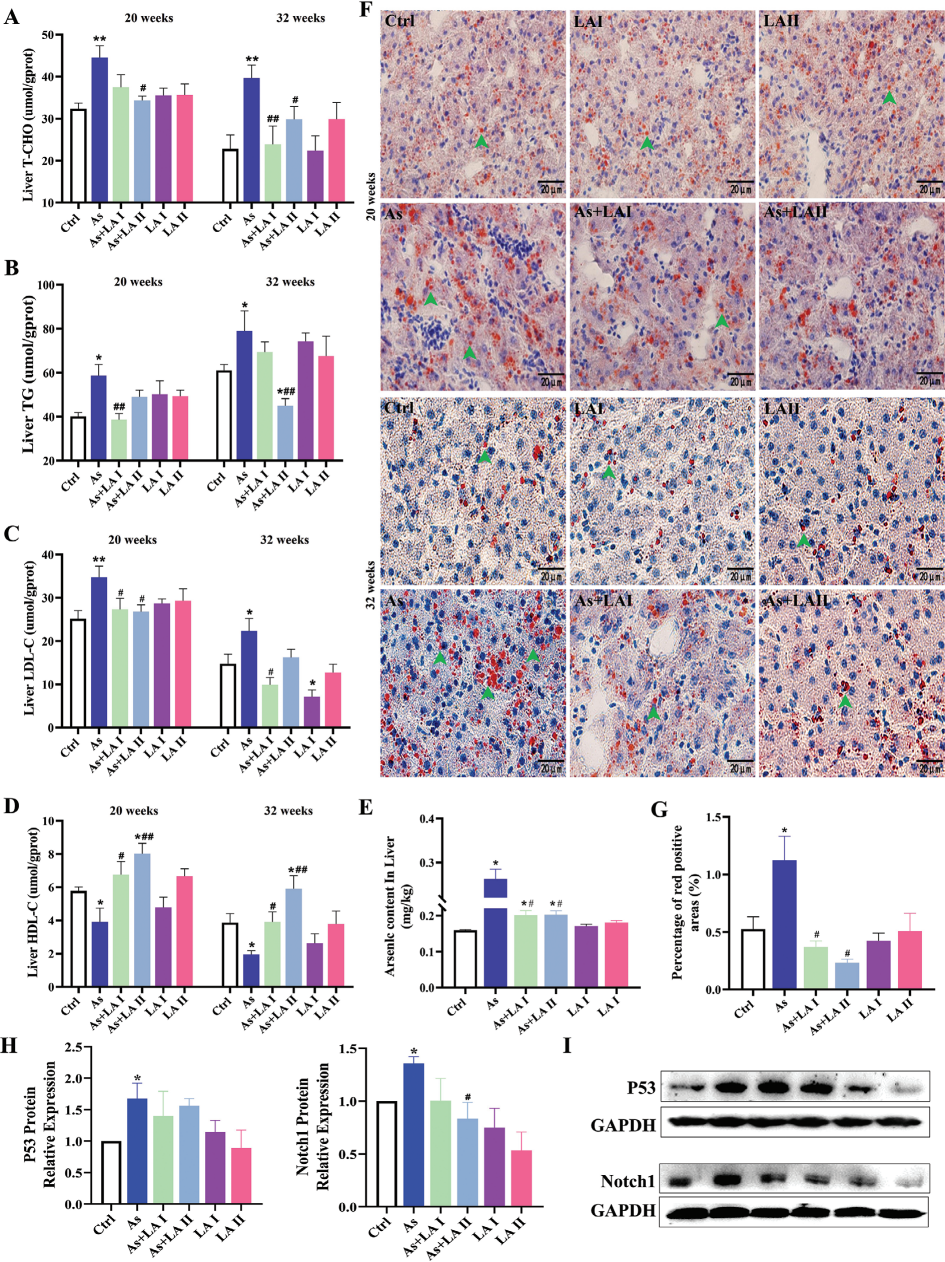
Effects of As exposure and LA intervention on lipid metabolism in the chicken liver. A–D) Detection results of the liver lipid metabolism index (T‐CHO, TG, LDL‐C, and HDL‐C), *n* = 6. E) Detection results of the liver As content, *n* = 6. F) Oil red O staining results (400×, green arrows: lipid droplets). G) Statistical results of Oil Red O staining, *n* = 6. H,I) Detection results of P53 and Notch1 protein expression levels, *n* = 3. ^*^ *P* < 0.05, ^**^ *P* < 0.01, versus Ctrl; ^#^ *P* < 0.05, ^##^ *P* < 0.01, versus As.

To further validate the hepatotoxic mechanism of As and the hepatoprotective mechanism of LA, this study used Oil Red O staining, lipid metabolism‐related indicators detection, RT‐PCR, and western blotting to assess the effects of As and/or LA on hepatic lipid metabolism. The results showed that As exposure significantly promoted the accumulation of TG, total cholesterol (T‐CHO), and low‐density lipoprotein (LDL) in the liver and decreased the level of high‐density lipoprotein (HDL) after 20 and 32 weeks' treatment. However, the addition of LA effectively reversed this trend, significantly reducing TG, T‐CHO and LDL levels and increasing HDL levels, among which the effect of 400 mg Kg^−1^ LA intervention group was particularly significant (*P* < 0.05, *P* < 0.01, **Figure** **4**A–D). Consistent with lipid detection results, Oil Red O staining results showed that the lipid (red positive area) content and the number of lipid droplets were increased in the liver after 20 and 32 weeks treatment, while LA intervention significantly reduced liver lipid content (Figure 4F). These findings suggested that LA could alleviate As‐induced liver damage by decreasing hepatic lipid content, with effects becoming more pronounced at 32 weeks.

Considering that As‐induced liver damage was more serious, and LA has a better effect in alleviating As‐induced liver function damage, tissue morphology damage, lipid metabolism disorders at 32 weeks. Therefore, the subsequent studies focused on the specific mechanism of LA in improving As‐induced liver damage at 32 weeks. Statistical analyses of Oil Red O staining and liver As content showed that both the red positive area and liver As content were significantly elevated after As exposure, while LA intervention effectively mitigated these changes (*P* < 0.05, *P* < 0.01, Figure 4E,G). Based on the results from network toxicology and network pharmacology, as well as supporting literature, it was identified that the P53 and Notch signaling pathways play a pivotal role in the amelioration of As‐induced hepatic lipid dysregulation by LA. Further detections of the key genes revealed that As exposure significantly elevated the protein expression levels of P53 and Notch1 in the liver, whereas intervention with LA effectively reduced the expression of both proteins (*P* < 0.05, Figure 4H,I). These results indicated that LA, especially 400 mg kg^−1^, had a beneficial effect on alleviating liver lipid metabolism disorders induced by As exposure, which was regulated by P53 and Notch pathway.

### 2.5 LA Mitigated the Inhibitory Effect of As on the Peroxisomal β‐Oxidation in the Liver

**Figure 5:**
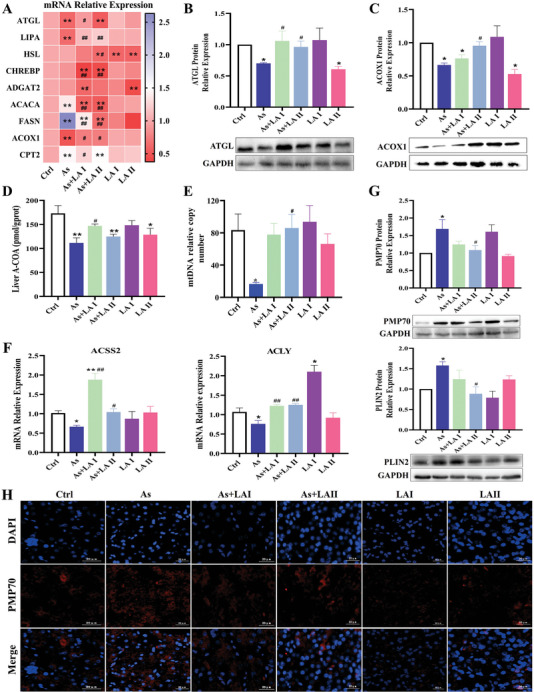
Effects of As exposure and LA intervention on the peroxisomal β‐oxidation in the chicken liver. A–C) The mRNA (*n* = 6)and protein (*n* = 3) expression levels of the lipid metabolism key genes detection results. D) Acetyl CoA detection results, *n* = 6. E) Results of mtDNA detection, *n* = 6. F) The mRNA expression levels of the fatty acid β‐oxidation key genes detection results, *n* = 6. G) The protein expression levels of the peroxisomal β‐oxidation key genes detection results, *n* = 3. H) Peroxisome immunofluorescence labeling results (400×). ^*^ *P* < 0.05, ^**^ *P* < 0.01, versus Ctrl; ^#^ *P* < 0.05, ^##^ *P* < 0.01, versus As.

To further investigate the effects of As and LA on hepatic lipid synthesis, degradation, and oxidation, the mRNA and protein expression levels of key genes involved in lipid synthesis (CHREBP, ADGAT2, ACACA, and FASN), lipid decomposition (ATGL, LIPA, HSL), and lipid oxidation (ACOX1, CPT2) were analyzed. The results indicated that As increased the mRNA expression of de novo lipid synthesis key genes (ACACA and FASN), and decreased the mRNA expression of lipid decomposition (ATGL, LIPA) and lipid oxidation (ACOX1, CPT2) key genes (*P* < 0.05, *P* < 0.01, Figure 4H). However, after intervention with LA, the mRNA expression levels of ATGL, LIPA, and ACOX1 were significantly increased, and the mRNA expression levels of ACACA, FASN, and CPT2 were considerably lower than those of the As group (*P* < 0.05, *P* < 0.01, **Figure** **5**A). Consistent with the mRNA detection results, the protein expression levels of ATGL and ACOX1 were remarkably decreased in the As group, but significantly increased after LA intervention (*P* < 0.05, Figure 5B,C).

Mitochondrial and peroxisome β‐oxidation are the central biological processes in lipid decomposition. The detection results showed that As treatment significantly reduced the content of acetyl‐CoA and mtDNA, while the addition of LA, effectively countered this decrease (*P* < 0.05, *P* < 0.01, Figure 5D,E). Moreover, As obviously decreased the mRNA expression levels of acetyl‐CoA synthesis key genes (ACSS2 and ACLY) in the liver, while LA intervention notably upregulated the mRNA expression of ACSS2 and ACLY (*P* < 0.05, *P* < 0.01, Figure 5F), suggesting that LA exerted a hepatoprotective effect by regulating the acetyl‐CoA metabolic pathway and resisting mitochondrial damage. Additionally, as shown in Figure 5G, LA intervention significantly reduced the As‐induced increase in the protein expression levels (*P* < 0.05) of PMP70 (peroxisome surface receptor) and PLIN2 (lipid droplet surface receptor). Further immunofluorescence staining using PMP70 to mark peroxisomes revealed that As treatment increased peroxisomes in the liver, while LA intervention was effective in reversing this change (Figure 5H), indicating that peroxisomal β‐oxidation was involved in the process by which LA alleviated As‐induced lipid deposition.

### 2.6 LA Reduced As‐Induced Hepatocyte Lipophagy

**Figure 6:**
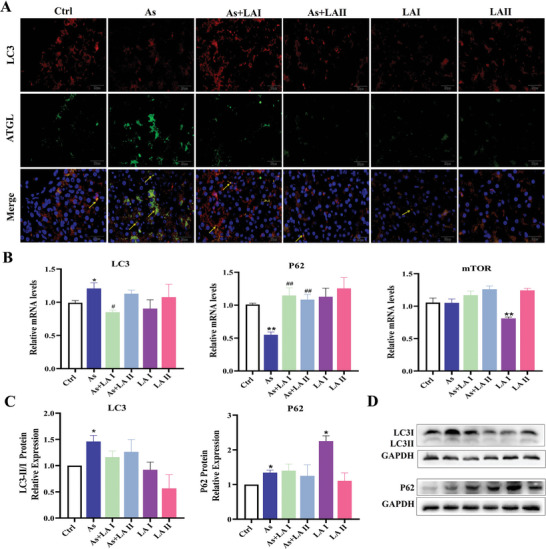
Effects of As exposure and LA intervention on the lipophagy in the chicken liver. A) Immunofluorescence colocalization lipophagy detection results (400×, ATGL: lipid droplets; LC3: lysosomes, yellow arrows: lipolysosome). B) Detection results of the mRNA expression levels of the lipophagy key genes, *n* = 6. C,D) Detection results of the protein expression levels of the lipophagy key genes in the lipid droplets, *n* = 3. ^*^ *P* < 0.05, ^**^ *P* < 0.01, versus Ctrl; ^#^ *P* < 0.05, ^##^ *P* < 0.01, versus As.

Immunofluorescence staining, RT‐PCR, and western blotting were employed to further explore the role of lipophagy in the LA‐mediated improvement of As‐induced hepatic lipid deposition. In the immunofluorescence experiments, ATGL and LC3 were used to locate lipid droplets and autophagosomes, respectively, thereby co‐localizing lipophagy. As shown in **Figure** **6**A, the fluorescence intensity of lipophagy in the As group was enhanced compared to that in the Ctrl group, indicating an increase in lipophagy. In contrast, the fluorescence intensity of the LA intervention group was diminished compared to the As group. The expression levels of key autophagy genes in lipid droplets revealed that compared with the Ctrl group, the mRNA expression of LC3 in the As group was significantly increased (*P* < 0.05), and the mRNA expression of P62 was significantly decreased (*P* < 0.05, *P* < 0.01, Figure 6B). However, compared with the As group, the changes in the mRNA expression levels of LC3 and P62 were alleviated in the LA intervention group (*P* < 0.05, Figure 6B), with no significant differences compared to the Ctrl group. Similar to the gene results, western blotting results showed that compared with the Ctrl group, the protein expression of LC3 and P62 was significantly increased in the As group, while the protein expression levels of LC3 and P62 in the LA intervention group were lower than those in the As group, and there was no significant difference between them and the Ctrl group (*P* < 0.05, Figure 6C,D). The above results indicated that As exposure induced hepatocyte damage by increasing lipophagy, whereas LA reduced lipophagy and alleviated this damage in the hepatocytes.

### 2.7 LA improved As‐Induced Hepatocellular Injury and Lipid Metabolism Disorders, and TRCDA Inhibited Peroxisomal β‐Oxidation In Vitro

**Figure 7:**
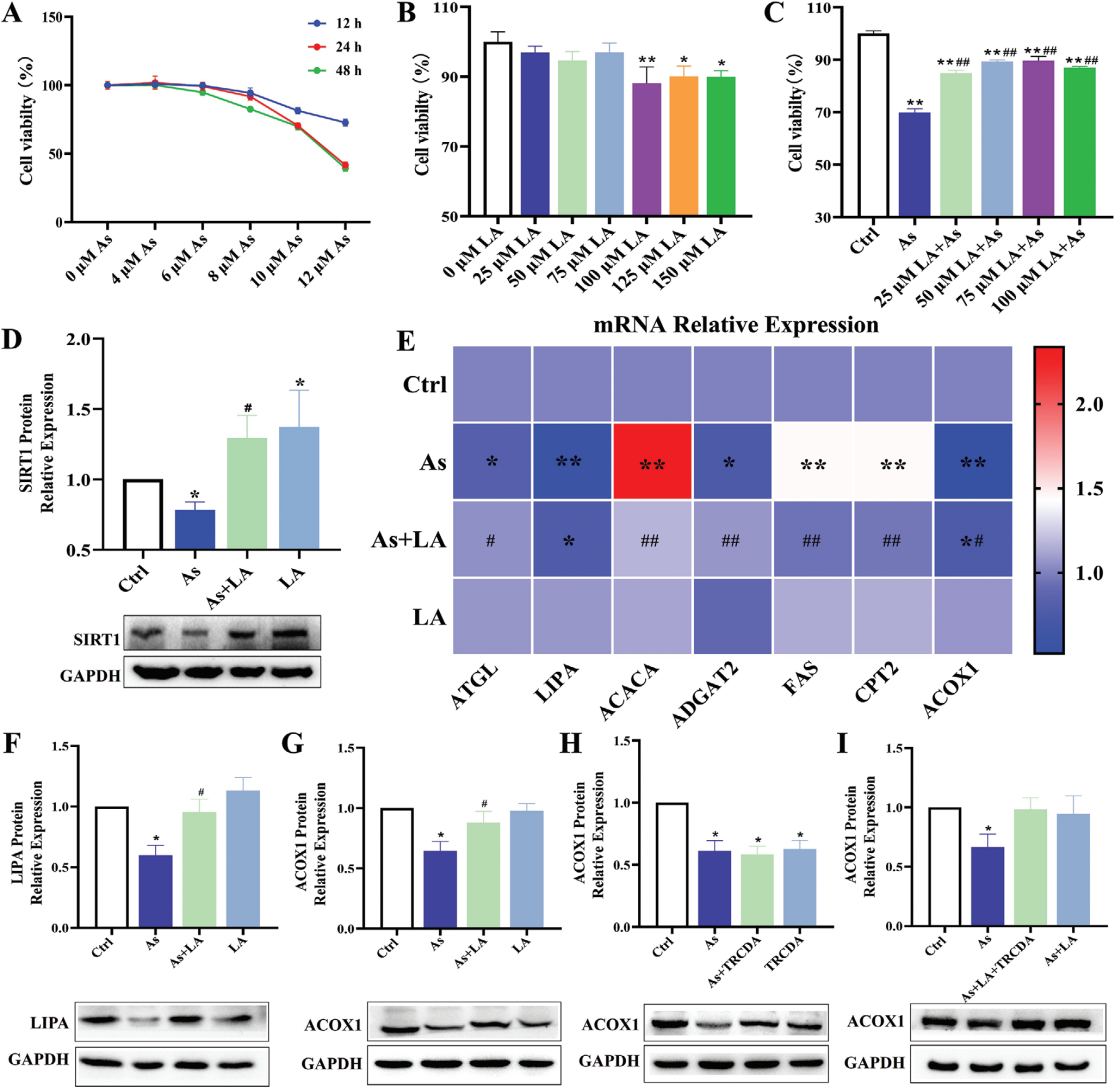
Establishment of the mechanism research model of chicken liver cell line (LMH cell line). A) Statistical results of cell proliferation rates under As treatment at different concentrations and for different times, *n* = 6. B) Statistical results of cell proliferation rate under treatment with different concentrations of LA, *n* = 6. C) Statistical results of cell proliferation rate under combined treatment with As and different concentrations of LA, *n* = 6. D) The protein expression levels of SIRT1 detection results, *n* = 3. E) Statistical results of the mRNA expression levels of the lipid metabolism key genes under As and/or LA treatment, *n* = 6. F) Statistical results of the protein expression level of LIPA under As and/or LA treatment, *n* = 3. G–I) Statistical results of ACOX1 protein expression levels in the different treatment groups, *n* = 3. ^*^ *P* < 0.05, ^**^ *P* < 0.01, versus Ctrl; ^#^ *P* < 0.05, ^##^ *P* < 0.01, versus As.

To further verify the protective mechanism of LA against As‐induced liver damage in a cellular model, this study initially used CCK‐8 experiments to assess the effects of As at varying concentrations and exposure durations and the impact of LA at different concentrations on the proliferation rate of LMH cells. As shown in **Figure** **7**A,B, with increasing concentrations of As and extended treatment durations, the proliferation rate of hepatocytes significantly decreased. In addition, as the LA concentration increased, the cells' proliferation rate exhibited a corresponding decrease (*P* < 0.05, *P* < 0.01). However, intervention with 25, 50, 75, and 100 µm LA significantly alleviated the decline in the hepatocytes proliferation rate caused by As treatment (69.89%), with corresponding proliferation rates of 84.92%, 89.29%, 89.58%, and 86.97%, respectively (*P* < 0.05, *P* < 0.01, Figure 7C). Based on the above results, this study determined that the treatment concentration of As was 10 µmol L^−1^, the treatment concentration of LA was 25 µmol L^−1^, and the treatment time was 24 h in the subsequent cell model research.

SIRT1, a key target protein of LA, was found to be downregulated by As, whereas LA effectively upregulated SIRT1 expression, which was consistent with in vivo findings (*P* < 0.05, Figure 7D). In addition, this study measured the mRNA and protein expression levels of lipolysis key genes to verify the alleviating effect of LA on As‐induced liver lipid metabolism disorders in the cellular model. The results showed that As treatment increased the mRNA expression of ACACA, FAS, and CPT2, while decreased the mRNA expression of ATGL, LIPA, ADGAT2, and ACOX1 in the hepatocytes. However, the combined treatment of As and LA ameliorated these alterations induced by As (*P* < 0.05, *P* < 0.01, Figure 7E). Moreover, consistent with the mRNA detection results, As reduced the protein expression of LIPA and ACOX1, whereas LA intervention increased the expression level of the protein in hepatocytes (*P* < 0.05, Figure 7F,G), suggesting that LA exerted a similar effect on improving lipid metabolism disorders at the cellular level as observed in vivo.

To further investigate the role of peroxisomal β‐oxidation in LA improving As‐induced liver lipid metabolism disorders, this study used TRCDA, an inhibitor of ACOX1, which is a key protein in the peroxisomal fatty acid β‐oxidation pathway, to establish a mechanical model of peroxisomal β‐oxidation. The ACOX1 protein detection results demonstrated that LA intervention increased the expression of ACOX1 protein, which was decreased by As treatment, while TRCDA treatment further reduced the expression of ACOX1 protein in hepatocytes (*P* < 0.05, *P* < 0.01, Figure 7H,I). These findings indicated that TRCDA inhibited peroxisomal β‐oxidation, which was implicated in the regulation of As‐induced liver toxicity and the protective effect of LA on the liver.

### 2.8 TRCDA Promoted As‐Induced Lipid Metabolism Disorders and Lipophagy While Reducing the Efficacy of LA in Improving Both Conditions

**Figure 8:**
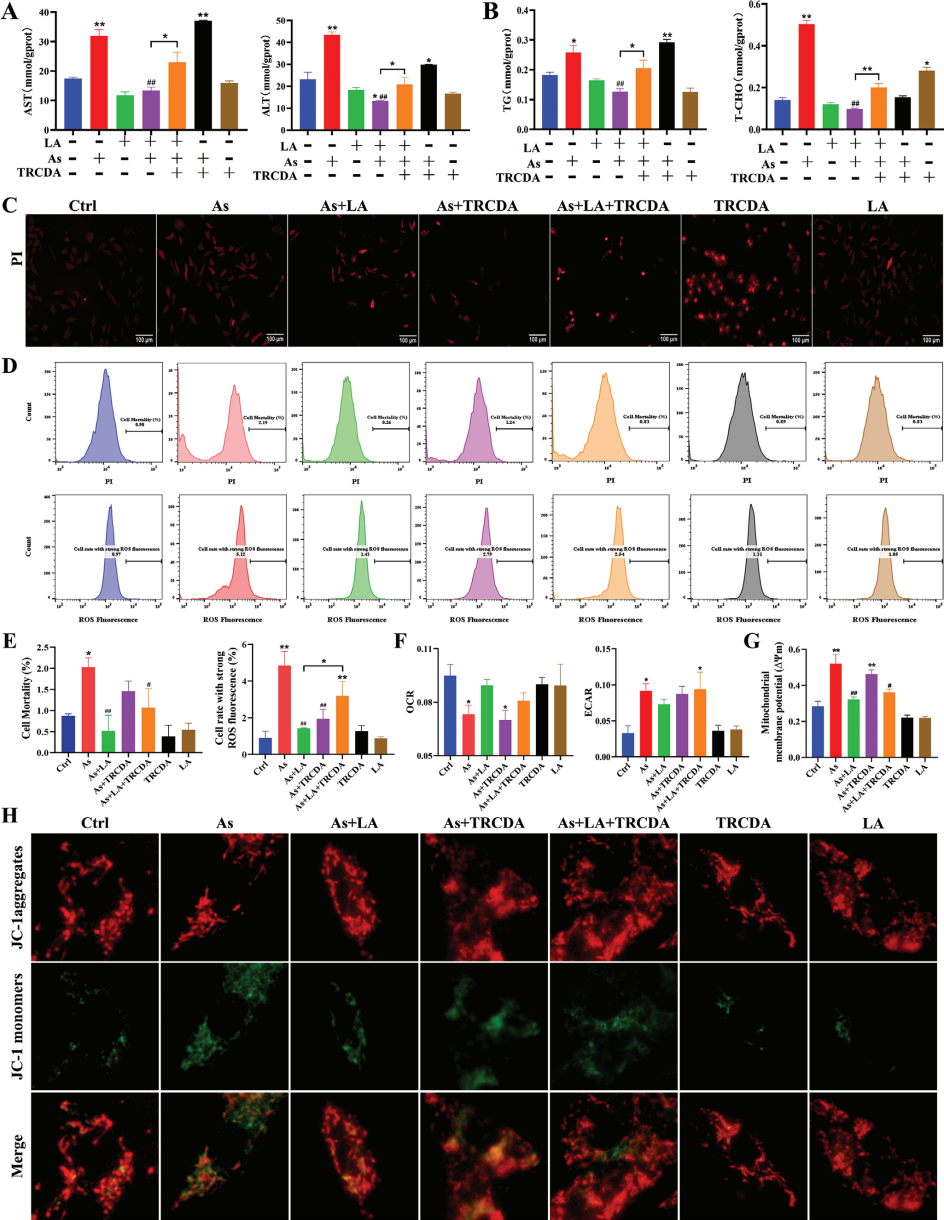
Effects of TRCDA treatment on hepatocyte function, lipid metabolism, ROS content, and cell death under As and/or LA exposure. A) Detection results of hepatocyte function indexes, *n* = 6. B) Detection results of lipid metabolism indexes, *n* = 6. C) PI fluorescence staining detection results (400×). D,E) PI and H2DCFDA fluorescence staining flow cytometry detection results, *n* = 6. F) OCR and ECAR detection results, *n* = 4. G) Mitochondrial membrane potential detection results, *n* = 6. H) JC‐1 fluorescence staining detection results.^*^ *P* < 0.05, ^**^ *P* < 0.01, versus Ctrl; ^#^ *P* < 0.05, ^##^ *P* < 0.01, versus As.

Hepatocyte function and lipid content index detection, fluorescent staining, and flow cytometry were employed to detect the effect of peroxisomal β‐oxidation inhibition on LA's capacity to ameliorate As‐induced hepatocyte damage and lipid metabolism disorders. As shown in **Figure** **8**, As treatment significantly increased the liver function indicators (AST and ALT, Figure 8A), lipid metabolism indicators (TG and T‐CHO, Figure 8B), cell death rate (Figure 8C–E), ROS content (Figure 8D,E), extracellular acidification rate (ECAR, Figure 8F), mitochondrial membrane depolarization rate (Figure 8G), but increased the oxygen consumption rate (OCR, Figure 8F) in the hepatocytes (*P* < 0.05, *P* < 0.01). Conversely, the contents of AST, ALT, TG, T‐CHO, ROS and the rate of cell death and mitochondrial membrane depolarization were significantly decreased in the As+LA group (*P* < 0.05, *P* < 0.01), and the OCR and ECAR of the As+LA group were not significantly different from the Ctrl group. Furthermore, TRCDA treatment inhibited the alleviating effects of LA on the aforementioned indicators altered by As, and tended to exacerbate the As‐induced increases in AST and TG levels (*P* < 0.05). These results further validated the protective effect of LA on As‐induced hepatic lipid metabolism disorders and mitochondrial damage, and confirmed the regulatory role of peroxisomal β‐oxidation in this process.

**Figure 9:**
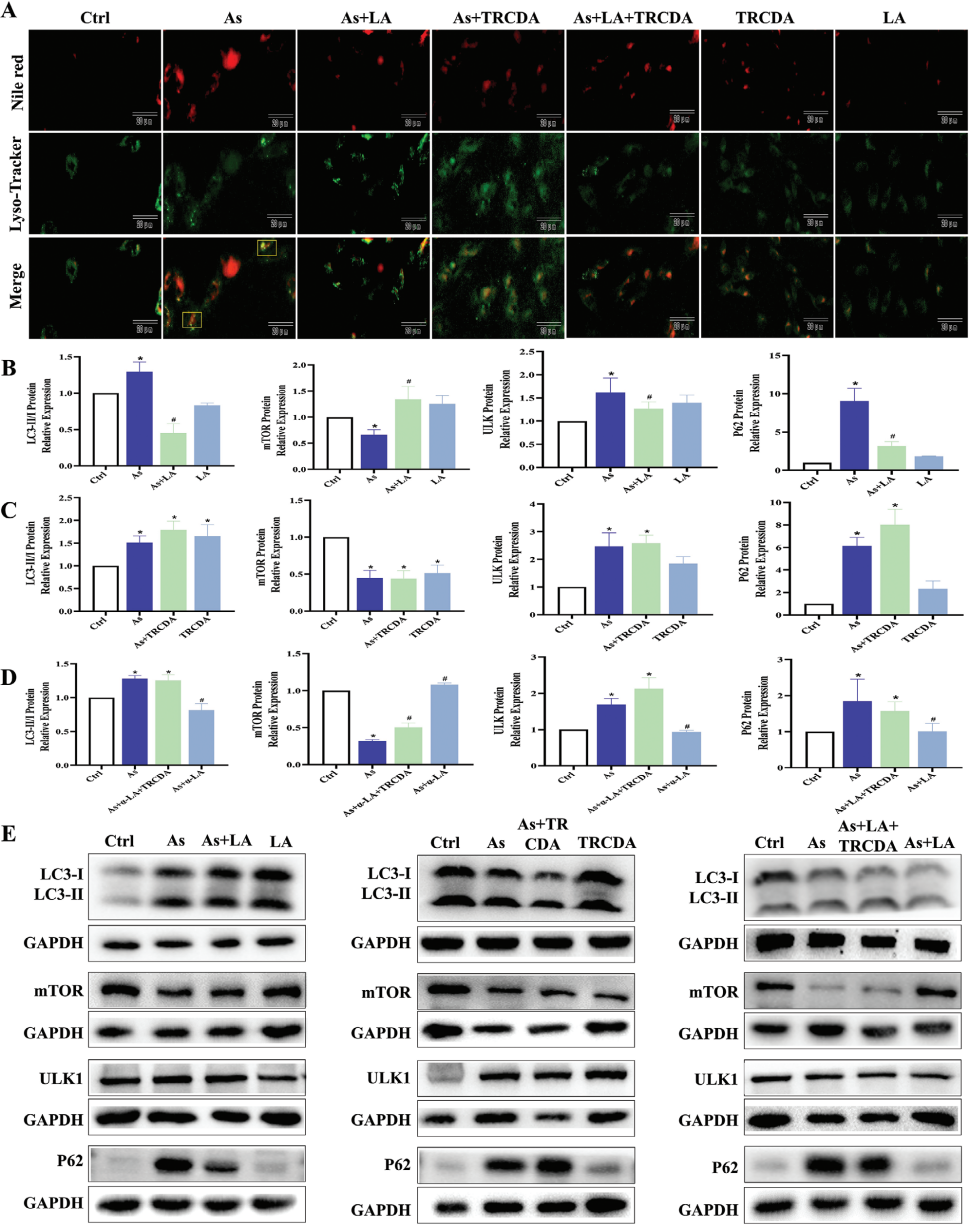
Effects of TRCDA treatment on the hepatocyte lipophagy under As and/or LA exposure. A) Fluorescence colocalization lipophagy detection results (400×, Nile red: lipid droplets; Lyso‐Tracker: Lysosome, yellow box: lipolysosome). B–E) Detection results of the protein expression levels of lipophagy key genes in the lipid droplets under different treatment groups, *n* = 3. ^*^ *P* < 0.05, versus Ctrl; ^#^ *P* < 0.05, versus As.

This study also investigated the regulatory role of peroxisomal β‐oxidation on lipophagy in the context of LA's improvement of As‐induced liver damage. The results showed that As treatment significantly increased the intensity and number of lipophagy fluorescence spots in the hepatocytes, elevated the protein expression levels of LC3, ULK, and P62, and reduced the protein expression level of mTOR in the hepatocyte lipid droplets, but LA intervention alleviated the above changes induced by As (P < 0.05, **Figure** **9**A–E). Additionally, TRCDA treatment further promoted the changes in As‐induced lipophagy‐related indicators, while inhibiting the ameliorative effect of LA on As‐induced lipophagy (*P* < 0.05, Figure 9A–E). These results suggested that peroxisomal β‐oxidation regulated lipophagy and played an essential role in improving As‐induced liver injury by LA.

## 3 Discussion

As pollution, driven by human activities such as ore mining, metal smelting, fuel combustion, and pesticide use, has become increasingly severe and has attracted significant attention from toxicology researchers.^[^ ^6^, ^30^ ^]^ The mechanisms of As toxicity are complex, affecting many tissues and organs, which seriously endangers human health and the development of animal husbandry.^[^ ^31^, ^32^, ^33^ ^]^ As targets the liver, and long‐term exposure impairs normal metabolic function, induces oxidative stress and inflammation, and causes structural and functional damage.^[^ ^34^ ^]^ To comprehensively analyze As‐induced liver toxicity and develop effective prevention strategies, this study utilized network toxicology analysis. The analysis revealed that As mainly affects the liver's biological processes such as oxidative stress, proliferation, differentiation, apoptosis, autophagy, lipid metabolism and inflammation by regulating related pathways. Further analysis found that oxidative stress and lipid metabolism disorders are central to arsenic‐induced liver injury, which not only directly damages hepatocytes but also contributes to the development of liver injury by affecting cell proliferation, differentiation, apoptosis, autophagy and other processes.^[^ ^35^, ^36^ ^]^ LA, a natural antioxidant whose protective mechanisms overlap with As's hepatotoxic pathways, has been shown to protect against severe liver diseases.^[^ ^26^, ^35^ ^]^ Therefore, this study established a chicken model of LA intervention in As exposure to further verify and explore the hepatoprotective effect and mechanism of LA on As‐induced liver damage. Although chickens serve as an agricultural animal model, the metabolic pathways and cellular mechanisms involved in As toxicity and the hepatoprotective effects of LA show significant similarities with those in humans and other mammals. Therefore, the findings obtained from the chicken model can provide a valuable reference for understanding the mechanism of arsenic‐induced liver injury and the hepatoprotective application of α‐lipoic acid in humans and other organisms. Moreover, the arsenic dosage used in this study closely approximates the levels that chickens in arsenic‐contaminated regions might ingest through polluted water or contaminated feed. Hence, the model not only has strong rationality, but also the results of the study have strong and wide applicability.

The liver is the main target organ for As storage and metabolism.^[^ ^37^ ^]^ Existing evidence showed that trace As stimulated growth and protein synthesis, but excessive As led to growth stunting, liver atrophy, weight loss, inflammatory infiltration, fatty degeneration, tissue disorder, necrosis, apoptosis, and elevated liver enzymes AST and ALT.^[^ ^34^ ^]^ In this study, As significantly affected body weight and liver function in chickens, but liver tolerance attenuated the effects of As on body weight,^[^ ^38^, ^39^ ^]^ especially after long‐term exposure. In addition, LA alone had no effect on body weight or liver function, but co‐exposure with As showed potential to mitigate As toxicity in chickens. These findings are similar to those of studies using mice and humans as research models. To further explore LA's hepatoprotective mechanisms, network pharmacology was used to analyze its biological characteristics, revealing that LA mainly ameliorates liver damage by regulating lipid metabolism. Therefore, this study further verified the effects of As and LA on liver lipid metabolism.

Lipid metabolism in the liver is essential for maintaining the body's lipid balance and normal physiological functions.^[^ ^40^ ^]^ It involves the synthesis and catabolism of phospholipids, CHOL, TG, and FFA, and lipoprotein metabolism for efficient lipid transport and utilization. Studies have shown that As can cause lipid metabolism disorders in the liver.^[^ ^41^, ^42^ ^]^ For example, Ditzel et al. found that prenatal and early postnatal As exposure induced mice liver fatty degeneration, significantly increasing TG, T‐CHO and FFA levels.^[^ ^43^ ^]^ Similarly, in the present study, As exposure caused lipid accumulation, vacuolar degeneration, and increased lipid droplets in the liver. TG and T‐CHO are important indicators of liver lipid metabolism.^[^ ^40^ ^]^ As exposure increased TG and T‐CHO levels, promoted de novo lipogenesis, and decreased lipolysis and fatty acid oxidation gene expression, further confirming the induction effect of As on hepatic lipid metabolism disorders. However, LA intervention alleviated these As‐induced changes. In human NAFLD studies, LA ameliorated liver injury by modulating lipid metabolism, which corroborates the results of the present study. Combined with the network pharmacology, network toxicology and related literature, it is found that SIRT1 served as a crucial target of LA, and the P53 and Notch signaling pathways played significant roles in the effects of LA and As on hepatic lipid metabolism. Further investigations confirmed that As exposure inhibited the expression of SIRT1 and activated the P53 and Notch pathways in the liver, whereas LA could bind to SIRT1, enhanced the expression and activity of SIRT1, and suppressed the P53 and Notch pathways. Previous studies have shown that SIRT1, through its deacetylation activity, modulated transcription factors associated with adipocyte differentiation, thereby inhibiting adipocyte differentiation.^[^ ^44^ ^]^ Additionally, SIRT1 activated PGC‐1α to promote fatty acid oxidation, reducing intracellular fatty acid accumulation.^[^ ^44^ ^]^ Meanwhile, the P53 and Notch pathways are crucial regulators of hepatic lipid metabolic dysregulation. The P53 pathway modulates the expression of genes involved in lipid synthesis and participates in the regulation of fatty acid oxidation, and the Notch1 pathway promotes adipocyte differentiation and leads to the dysregulation of lipid metabolism genes by interactions with various transcription factors.^[^ ^45^ ^]^ Based on these findings, it can be inferred that As inhibited SIRT1 expression and activated the P53 and Notch pathways, resulting in hepatic lipid metabolic disturbances. In contrast, LA targeted SIRT1, enhancing its expression and activity, thereby exhibiting antioxidant properties, modulating lipid metabolic pathways, reducing hepatic fat synthesis and accumulation, promoting lipolysis, and effectively alleviating hepatic fat accumulation and lipid peroxidation.^[^ ^22^, ^46^ ^]^ Peroxisomal β‐oxidation and lipophagy are essential processes in lipid metabolism.^[^ ^12^, ^47^ ^]^ Hence, this study further investigated their roles in As‐induced hepatotoxicity and LA's hepatoprotective effects.

Peroxisomes, unique organelles in eukaryotic cells, are important regulators of liver lipid metabolism homeostasis.^[^ ^10^ ^]^ They are responsible for removing harmful hydrogen peroxide to maintain cellular redox balance, as well as for the β‐oxidation of very long‐chain fatty acids, converting them into acetyl‐CoA through a series of enzymatic reactions.^[^ ^11^ ^]^ In this study, As exposure resulted in decreased expression of the peroxisomal β‐oxidation enzyme ACOX1, disrupting peroxisomal β‐oxidation. Conversely, As promoted mitochondrial fatty acid β‐oxidation, likely as a compensatory mechanism to mitigate the impaired peroxisomal β‐oxidation, with LA supplementation alleviating this effect.^[^ ^48^ ^]^ Acetyl‐CoA is a key product of peroxisomal β‐oxidation, and LA could reverse the decrease in acetyl‐CoA levels caused by As exposure. Cytosolic acetyl‐CoA primarily derives from two sources: ACLY and ACSS2.^[^ ^49^ ^]^ In this study, LA administration improved the mRNA expression of ACLY and ACSS2, confirming that both As and LA influence acetyl‐CoA levels through peroxisomal β‐oxidation. Additionally, As exposure elevated the expression of PMP70, a receptor protein on peroxisomes, indicating an increase in peroxisome quantity in the liver. This suggested an adaptive response by hepatocytes to As exposure, aimed at enhancing peroxisomal function or stability. However, this increase was insufficient to fully counteract As's inhibitory effect on peroxisomal β‐oxidation, resulting in elevated expression of PLIN2 and subsequent lipid droplet accumulation.^[^ ^50^ ^]^ LA intervention could regulate these processes, enhancing As‐induced peroxisomal β‐oxidation and alleviating lipid metabolism disorders. Furthermore, the accumulation of lipid droplets may trigger enhanced lipophagy, with excessive lipophagy potentially exacerbating cell damage.

Lipophagy, a selective autophagy process, specifically recognize and degrade lipid droplets in the liver, thereby regulating lipid homeostasis and reducing lipid accumulation.^[^ ^16^, ^51^ ^]^ However, abnormal enhancement of lipophagy can result in organelle damage, lipid metabolism imbalance, oxidative stress, and inflammatory response, ultimately harming cells.^[^ ^16^, ^52^ ^]^ For instance, Yu et al. demonstrated that a high‐fat diet could induce nonalcoholic fatty liver disease and liver aging via lipophagy in both rats and HepG2 cells.^[^ ^53^ ^]^ Additionally, Yoshito et al. found that enhancing lipophagy effectively alleviated steatosis and nonalcoholic steatohepatitis in mice.^[^ ^16^ ^]^ These findings suggested that the accumulation of lipid droplets triggers enhanced lipophagy. In this study, As led to lipid droplets accumulation and increased levels of LC3 and P62 in the lipid droplets, indicating enhanced lipophagy. Notably, LA intervention reduced lipophagy in hepatocytes, as further validated by immunofluorescence colocalization. The enhancement of lipophagy may represent an adaptive response of cells to As toxicity, aimed at mitigating damage from lipid overload. However, LA intervention appears to alleviate lipid droplet deposition and lipophagy through its antioxidant effects, enhancing peroxisomal β‐oxidation and regulating lipid metabolism pathways, thus reducing As‐induced cellular damage.^[^ ^17^, ^54^, ^55^ ^]^ To further investigate the mechanism of peroxisomal β‐oxidation and lipophagy in As hepatotoxicity and LA hepatoprotective effect, this study established a cell research model.

Peroxisomal β‐oxidation and lipophagy are key processes in liver lipid catabolism.^[^ ^10^, ^16^, ^55^ ^]^ Although the regulatory relationship between these two processes remains unclear, studies have indicated that acetyl‐CoA produced by peroxisomal β‐oxidation can inhibit autophagy and promote fatty degeneration.^[^ ^56^ ^]^ In this study, treatment with the peroxisomal β‐oxidation inhibitor TRCDA exacerbated the inhibitory effects of As on β‐oxidation, diminished the promoting effects of LA on β‐oxidation, and attenuated the protective effects of LA against As‐induced liver function damage and lipid deposition. These findings further corroborated that LA mitigated As‐induced liver lipid metabolism disorders by enhancing oxidase β‐oxidation. Additionally, this study observed that TRCDA treatment intensified the promoting effect of As on lipophagy while concurrently reducing the inhibitory effects of LA on lipophagy, which confirmed the regulatory effect of peroxisomal β‐oxidation on lipophagy in the improvement of As‐induced liver lipid metabolism disorders by LA.

Interestingly, ROS, a byproduct of peroxisomal β‐oxidation, was elevated despite As inhibiting peroxisomal β‐oxidation. Mitochondria, the primary site of ROS production, exhibit decreased membrane potential and impaired oxidative phosphorylation when it is damaged, resulting in reduced oxygen consumption, as reflected by a OCR decrease.^[^ ^57^ ^]^ Moreover, mitochondrial dysfunction leads to decreased ATP synthesis, which in turn activates glycolysis, thereby increasing lactate production, which accelerates extracellular acidification, as reflected by an ECAR increase.^[^ ^57^ ^]^ In this study, As exposure was shown to reduce mtDNA, mitochondrial membrane potential, and OCR, while increasing ECAR, indicating mitochondrial damage and impairment of oxidative phosphorylation and cellular glycolysis. Mitochondrial damage directly increases ROS content, which helps explain the elevated ROS levels after As exposure. However, LA intervention was found to reverse the mitochondrial damage induced by As exposure. The underlying mechanism may involve the toxic effects of As, which inhibited SIRT1 expression, activated the P53 and Notch pathways, damaged mitochondria, and inhibited peroxisomal β‐oxidase activity, thereby impairing fatty acid degradation and leading to lipid accumulation in hepatocytes.^[^ ^38^ ^]^ In response to this lipid overload, cells initiated the lipophagy mechanism to eliminate excess lipid droplets, and the excessive enhancement of lipophagy ultimately affected the proliferation and death of liver cells.^[^ ^7^, ^58^ ^]^ Conversely, as an antioxidant that promotes metabolic activity, LA targeted to SIRT1, effectively improved mitochondrial function, enhanced the β‐oxidation function of peroxisomes, and reduced the excessive accumulation of lipids in liver.^[^ ^59^ ^]^ This mechanism not only directly alleviates the lipid burden on the liver but also inhibits the excessive activation of lipophagy by restoring the balance of lipid metabolism, thereby effectively mitigating As‐induced liver lipid metabolite disorders and cell death.^[^ ^21^ ^]^ The key pathways and intrinsic mechanisms by which peroxisomal β‐oxidation regulates lipophagy will be further explored in future studies.

## 4 Conclusion

This study indicated that As inhibited SIRT1 expression, activated the P53 and Notch pathways, induced mitochondrial damage, and inhibited peroxisomal β‐oxidation, resulting in lipid deposition that enhanced lipophagy and led to structural and functional liver damage. However, LA intervention mitigated As‐induced liver damage by targeting SIRT1, ameliorating mitochondrial damage, enhancing β‐oxidation, regulating lipid metabolism, and inhibiting lipophagy, especially 400 mg kg^−1^. This study further clarified the regulatory effect of peroxisomal β‐oxidation on lipophagy in hepatocytes, and their underlying mechanisms in As‐induced hepatotoxicity and the hepatoprotective effect of LA. These findings provide a theoretical basis for LA as a potential therapeutic agent for As‐induced liver damage and offer new insights and directions for research in liver diseases.

## 5 Experimental Section

### Animals and Treatment

One hundred twenty Hailan Brown chicks (one day old, specifically pathogen‐free) were bought from a commercial hatchery (Jinzhong, China). After one‐week adaptation, the chicks were randomly divided into 6 groups: control group (Ctrl: ordinary chicken feed), As treatment group (As: As_2_O_3_), LA treatment groups (LAI: 200 mg kg^−1^ LA, LAII: 400 mg kg^−1^ LA), As and LA treatment groups (As+LAI: As_2_O_3_+200 mg kg^−1^ LA, As+LAII: As_2_O_3_+400 mg kg^−1^ LA), 20 chicks per group. The doses of As_2_O_3_ was 36 mg kg^−1^ (As: 27.27 mg kg^−1^), and the As_2_O_3_ and LA were added to the feed. (R)‐(+)‐α‐lipoic acid was used in this study and it was purchased from Aladdin Biochemical Technology Co., Ltd. (D118666, CAS: 1200‐22‐2, Shanghai, China). All chickens were fed a standard diet under standard conditions. At the 23rd and 35th weeks of rearing, 10 chickens in each group were euthanized, and the livers were collected for the detection of relevant indicators. This experiment was approved by the Animal Care and Use Committee of Shanxi Agricultural University (SXAU‐EAW‐2023C.QR.001011208).

### LMH Cell Culture, Passaging and Treatment

The LMH cell used in this study was a cell line frozen in the laboratory, which was commonly used for studying chicken liver diseases. After removing the frozen cells from liquid nitrogen, the cells were rapidly transferred to a 37 °C water bath for thawing. Then, the cells were centrifuged, the supernatant was discarded, and the complete culture medium was added. The mixture was gently resuspended and transferred to a culture flask placed in an incubator. When the cells grew to 80% density, they were passaged, and when the cells reached a sufficient number, they were grouped and treated according to the experimental requirements. As_2_O_3_ and LA were diluted with a complete culture medium.

### CCK‐8 Detection

CCK‐8 assays were used to assess the effects of different concentrations of As/LA on the proliferation rate of LMH cells and to determine the optimal treatment concentrations of As and LA in vitro. Briefly, 100 µL cell suspension was seeded into a 96‐well plate, and As and/or LA was added after 24 h (the final concentration of As was 0, 4, 6, 8, 10, and 12 µmol L^−1^, and the final concentration of LA was 0, 25, 50, 75, 100, 125, and 150 µmol L^−1^). After 12, 24, and 48 h of cell culture, 10 µL CCK‐8 (C0037, Beyotime, Shanghai) was added to each well and incubated for 2 h. The absorbance was recorded at 450 nm, and the proliferation rate of different treatment groups was calculated.

### Network Toxicology and Network Pharmacology

Network toxicology was used to analyze the potential mechanisms of As‐induced liver injury, and network pharmacology was used to analyze the potential protective effects of LA against As‐induced liver injury. Network toxicology: The keywords “Arsenic” and “liver injury” were searched in the CTD (https://ctdbase.org/) and GeneCards database (https://www.genecards.org/), respectively.^[^ ^60^ ^]^ The relevant genes searched from the two databases were intersected to obtain potential target genes for As‐induced liver damage, and the GO and KEGG enrichment analysis was performed in the Matescape (http://metascape.org/gp/index.html). Network pharmacology: Potential targets of LA were screened in the Swiss Target Prediction (http://swisstargetprediction.ch/) and TargetNet databases (http://targetnet.scbdd.com/), and the target genes searched from the two databases were combined.^[^ ^61^ ^]^ Then, the Matescape database and Cytoscape/Cluego (version 3.7.2, USA) were used to screen key target genes and conduct GO and KEGG enrichment analysis. In addition, molecular docking and visualization were performed using the SwissDock (https://www.swissdock.ch/) and UCSF Chimera software (version 1.13.1, USA) for further screening.

### Detection of As Content in the Liver and Feed

Inductively coupled plasma mass spectrometry (ICP‐MS) was used to determine the content of As ions in the liver and feed.^[^ ^62^ ^]^ Briefly, 1 g liver tissue or feed was weighed and dried to constant weight. Subsequently, it was finely ground into powder, and 100 mg of the powder was poured into a digestion tube containing 8 mL of concentrated nitric acid. The digestion tube was placed in a digestion apparatus for digestion according to conventional steps. After constant volume, the solution was filtered using a filter membrane and detected using an ICP‐MS (NexION 350, PerkinElmer, Waltham). Finally, the actual As content in the sample was calculated based on the standard curve.

### HE Staining

Liver morphological damage was evaluated using HE staining. After the liver was fixed, it was rinsed, dehydrated, transparent, wax‐impregnated, embedded, and 5 µm paraffin sections were made. The paraffin sections were routinely dewaxed and stained with eosin and hematoxylin, and the morphological structure of the liver was observed under an ordinary optical microscope (BX51, Olympus, Tokyo).

### Oil Red O Staining

Oil Red O staining was used to observe lipid content in the liver. Fresh liver tissue was embedded in OCT embedding medium, and 8 µm frozen sections were prepared. Then, the sections were washed with 60% isopropanol for 2 min and stained with Oil Red O for 15 min in the dark. Finally, after washing with 75% anhydrous ethanol, the sections were counterstained with hematoxylin and mounted with glycerol‐gelatin. Under the ordinary optical microscope, the lipid droplets appeared orange‐red and cell nuclei appeared blue.

### Detection of Liver Function and Lipid Metabolism‐Related Indicators

The liver function and lipid metabolism were observed by detecting the levels of liver function‐related indicators (ALT (C009‐2‐1), AST (C010‐2‐1)) and lipid metabolism‐related indicators (T‐CHO (A111‐1‐1), TG (A110‐1‐1), LDL‐C (A113‐1‐1), HDL‐C (A112‐1‐1)) in the liver and cells. These kits were purchase from Nanjing Jiancheng Bioengineering Institute (Nanjing). For liver samples, a 10× homogenate was prepared. For cell samples, cells were collected, disrupted by ultrasonic waves, and centrifuged to obtain the supernatant to obtain the cell test solution. Subsequently, the procedure was carried out according to the kits' instructions. Finally, the OD values were measured using a microplate reader (Varioskan Flas, Thermo Fisher, Waltham), and the results were analyzed.

### mtDNA Detection

Total DNA was extracted using a DNA extraction kit (D1700, Solarbio, Beijing) and quantified with a NanoDrop 2000 (Thermo Scientific, Massachusetts). The DNA was then diluted to a concentration of 150 ng µL^−1^. Primers were designed based on the chicken D‐loop (mitochondrial gene) and GCG (nuclear gene) sequences and synthesized. The primer sequences were provided in Table S1 (Supporting Information). RT‐PCR was performed using a RT‐PCR kit (RR420A, Takara Biomedical Technology) following the manufacturer's instructions. The relative level of mtDNA was calculated by 2^△CT^.

### Mitochondrial Membrane Potential Detection

Cells were seeded in 96‐well/12‐well plates and cultured for 24 h. Then, cells were treated according to the experimental grouping. After 24 h, the culture medium was replaced, and JC‐1 dye (C2006, Beyotime, Shanghai) was added at a final concentration of 10 µg mL^−1^. The cells were incubated at 37 °C for 20 min, after which culture medium was replaced. Mitochondrial membrane potential changes were detected using a multifunctional microplate reader or fluorescence microscope. At higher mitochondrial membrane potential, it appeared as J‐aggregates (525 nm/590 nm, red fluorescence), while at lower mitochondrial membrane potential, it appeared as monomer (490 nm/530 nm, green fluorescence). The ratio of green/red fluorescence was used to quantify the change in mitochondrial membrane potential.

### OCR and ECAR Detection

After treatment according to the experimental grouping, cells were cultured for 24 h, then the culture medium was discarded. OCR (E‐BC‐F068) and ECAR (E‐BC‐F069) were measured using corresponding assay kits (Elabscience, Wuhan) according to the manufacturer's instructions. The microplate was placed in a multifunctional microplate reader set at 37 °C with dynamic reading mode. For OCR detection, the excitation wavelength was set at 405 nm, the emission wavelength was set at 675 nm, and the detection was performed every 2 min for 90 min. For ECAR detection, the excitation wavelength was set at 490 nm and the emission wavelength was set at 535 nm, and the detection was performed every 2.5 min for 120 min. Finally, the fluorescence values were plotted against time, and OCR and ECAR were represented by the slopes of the curves.

### Lipophagy Detection

Liver samples were immunofluorescently double‐stained with autophagosome marker protein (LC3) and lipid droplet marker protein (ATGL) to co‐localize lipophagy in the hepatocytes. Paraffin‐embedded liver sections were deparaffinized and rehydrated using a standard protocol, and antigen retrieval was performed with citrate buffer for 10 min. Then, the sections were blocked with 5% BSA for 2 h and incubated overnight at 4 °C with primary antibodies LC3 (1:200, mouse‐derived, A17424, ABclonal Technology, Wuhan) and ATGL (1:200, rabbit‐derived, A6245, ABclonal Technology). After washing, the sections were incubated at room temperature with two fluorescent secondary antibodies for 2 h. Finally, the sections were mounted with an anti‐fade mounting medium containing DAPI and observed under a fluorescence microscope for imaging and colocalization analysis.

Cell samples were double‐stained with a lysosomal fluorescent probe (Lyso‐Tracker Green, C1047S, Beyotime) and lipid fluorescent probe (Nile Red, C2051S, Beyotime) to co‐localize lipophagy in the hepatocytes. After treating and culturing the cells according to the experimental requirements, the medium was replaced with fresh culture medium, and Lyso‐Tracker Green (final concentration 75 nmol L^−1^) and Nile Red (final concentration 1 µmol L^−1^) were added. Then, the cells were incubated at 37 °C for 30 min in the dark. After incubation, the cells were washed with PBS and observed under an inverted fluorescence microscope for imaging and colocalization analysis.

### Flow Cytometric Detection

Flow cytometry was used to detect cell apoptosis (PI, HY‐D0815, MedChemExpress, Shanghai) and reactive oxygen species (ROS, H2DCFDA, HY‐D0940, MedChemExpress). The cells were treated according to the experimental requirements. After 24 h of culture, the cells were collected, washed with PBS, and stained with 200 µL PI (concentration 50 µg mL^−1^) or H2DCFDA (concentration 5 mm) for 30 min. Subsequently, the cells were fixed with precooled 70% ethanol for 30 min, filtered through 200 mesh nylon cloth and loaded onto the machine. Finally, the cells' apoptosis and ROS levels were analyzed based on the experimental results.

### Western Blotting

100 mg liver tissue was homogenized in 1000 µL RIPA lysis buffer containing 10 µL PMSF, three samples per group. And the samples were centrifuged at 10 000 *g* for 15 min at 4 °C. For the detection of protein expression levels of lipophagy‐related genes, lipid droplets were extracted using a lipid droplet extraction kit (CBL‐MET‐5011, Cell Biolabs, San Diego) and then lipid droplet proteins were extracted. The protein concentration in the supernatant was determined using a BCA assay kit (AR1189, Boster Biological Technology, Wuhan). Then, 60 µg proteins were separated by electrophoresis on 8% and 12% SDS‐PAGE gels (80 V for 30 min, followed by 100 V for 90 min). The separated proteins were transferred onto NC membranes (350 mA for 1 h) and blocked with 5% nonfat dry milk for 2 h. The membranes were then incubated overnight at 4 °C with primary antibodies GAPDH (1:10000), P53 (1:2000), Notch1 (1:1000), SIRT1 (1:1000), PMP70 (1:1000), ATGL (1:1000), PLIN2 (1:1000), ACOX1 (1:3000), LC3B (1:1000), P62 (1:1000), mTOR (1:1000), and ULK1 (1:1000). GAPDH (bsm‐33033M), mTOR (bs‐1992R), ULK1 (bs‐3602R), and P62 (bs‐55207R) bought from Bioss (Beijing), and PMP70 (A4172), PLIN2 (D161460), P53 (10442‐1‐AP), Notch1 (10062‐2‐AP), SIRT1 (SIRT1), and ACOX1 (10957‐1‐AP) bought from ABclonal Technology, Sangon (Shanghai) and Proteintech (Wuhan), separately. After washing three times with TBST, the membranes were incubated with a secondary antibody (1:5000, RS0002, RS0001, ImmunoWay, Beijing) at 37 °C for 2 h. The signals were then developed using ECL chemiluminescence and analyzed with the FluorChem Q multifunction imaging system (Cell Biosciences, CA).

### RNA Extraction and RT‐PCR Detection

RT‐PCR was used to assess the relative mRNA expression levels of genes related to fatty acid metabolism in the liver. Total mRNA was extracted from 40 mg liver tissue using a Total RNA Extractor (B511311, Sangon, Shanghai), six samples per group. The mRNA concentration was measured using a NanoDrop 2000 and adjusted to 250 ng µL^−1^. The mRNA was then reverse‐transcribed into cDNA using the PrimeScript RT reagent kit (RR037A, Takara Biomedical Technology, Beijing). The relative mRNA expression levels were subsequently measured according to the protocol of the qRT‐PCR Kit. Primer sequences for GAPDH, ACOX1, ACACA, FASN, ATGL, LIPA, HSL, CHREBP, ADGAT2, LC3B, P62, and MTOR were provided in Table S1 (Supporting Information). The relative expression levels of the target genes were calculated using the 2^−△△CT^ method.

### Statistical Analysis

Data are presented as mean ± SEM and were analyzed using GraphPad Prism 8.0 software. Statistical comparisons were performed using One‐way ANOVA and multiple comparisons. The significant differences compared with the control group were marked with “*,” and the significant differences between the LA intervention groups and the As group were indicated by “#,” the LA alone treatment group was not compared with the As group. A *p*‐value less than 0.05 was considered statistically significant.

### Ethics Approval Statement

The animal experiments were approved by the Animal Care and Use Committee of Shanxi Agricultural University (SXAU‐EAW‐2023C.QR.001011208)

## Conflict of Interest

The authors declare no conflict of interest.

## Author Contributions

Y.Z.: formal analysis, supervision, methodology, writing‐original draft, and funding acquisition. M.G.: formal analysis, validation, methodology, and writing‐review & editing. T.P., C.S., and Y.C.: validation, writing‐review & editing. L.Z. and Y.L.: methodology. C.L. and J.W.: writing‐review & editing and supervision. J.Z.: project administration, supervision, funding acquisition, and writing‐review & editing.

## Supporting information



Supporting Information

## Data Availability

All data needed to evaluate the conclusions in the paper are present in the paper and/or the Supporting Information. There are no restrictions on the use of materials.
