# Supplementary material for: α‐Lipoic Acid Ameliorates Arsenic‐Induced Lipid Disorders by Promoting Peroxisomal β‐Oxidation and Reducing Lipophagy in Chicken Hepatocyte
Source: Adv Sci (Weinh). 2025 Jan 30;12(11):2413255. doi: 10.1002/advs.202413255 (PMC11923885; doi:10.1002/advs.202413255)
Supplement: Supplementary file 1 — Supporting Information [file ADVS-12-2413255-s001.docx]

**Supporting Information**

α-lipoic acid improves arsenic-induced liver lipid metabolism disorders by promoting peroxisomal β-oxidation and reducing lipophagy in chickens

*Yangfei Zhao^1*^, Mingyue Guo^1^, Ting Pei^1^, Chenqi Shang^1^, Yirong Chen^1^, Liying Zhao^1^, Yiguang Lu^1^, Chen Liang^2^, Jundong Wang^1^, Jianhai Zhang^1*^*

**Table S1 Primer Sequences for mRNA**

| **Name** | **Primer sequence** | **Accession No.** | **Product Size (bp)** |
| --- | --- | --- | --- |
| *GAPDH* | F: CAGAACATCATCCCAGCGTCCAC  R: CGGCAGGTCAGGTCAACAACAG | NM204305.2 | 134 |
| *ACLY* | F: TGCCATCCAGAACCGCTTCAAG  R:TTCACCACTAAACGCTCGCTCAG | XM040653073.2 | 105 |
| *AGPAT2* | F: CCATCATTGTGTCCAACCACCAGAG  R: CGGCGTACATCAGCTCCTTCTTG | XM040685441.2 | 105 |
| *HSL* | F: CATCCTGTCCGTCGATTACTCCTTG  R: CAGCAGTAGGCGTAGAAGCACTC | XM040695200.2 | 81 |
| *ACSS2* | F: GGACCGATAGCAACGCCTGATTAC  R: CCGCCTGGTGATCTTTCCTGAAC | XM046930956.1 | 78 |
| *LIPA* | F: TGGCTCACTACAACCAGTCTACTCC  R: AGTCCTGTCCACCAGTCCATATAGC | XM040675250.2 | 87 |
| *ACOX1* | F: AACAAGCCATCAGGAAGA  R: AAGGGTAGGGAGGAACAT | M001006205.2 | 152 |
| *CHREBP* | F: CTTTGACACACTGCACAGCC  R: TGATGACGCCGAAGATCCAG | XM046929861.1 | 320 |
| *ACACA* | F: CAGACGGTTGTGGTTGGCAGAG  R: TGGCGGGATCAGCAGGGATG | XM046929960.1 | 103 |
| *mTOR* | F: ACAACCACTGCTCGCCACAAT  R: CCATAGGATCGCCACACGGATT | NM018769.4 | 119 |
| *LC3* | F: GCATCCAAACAAAATCCCAGTC  R: AAGCCATCCTCATCCTTCTCCT | XM040688401.2 | 246 |
| *P62* | F: GACCCAGCCAAGACTACCAT  R: CAGAGGCATGTAGTTTCGGC | XM003642061.4 | 240 |
| *CPT2* | F: TTTGATGCCAGTGTAGAAGGAC  R: CAGTCTGATTGTATTGCCGAAG | NM001004142.2 | 124 |
| *FAS* | F: TTTGGTGGTTCGAGGTGGTA  R: CAAAGGTTGTATTTCGGGAGC | NM009068.3 | 81 |
| *D-loop* | F:ACCCCTGCCTGTAATGTACTTC | AM746040.1 | 183 |
|  | R:CACGGACTAAAGAGGGGAAGAT |  |  |
| *GCG* | F:GTGGAGGGCTGATAAAACACAAT | DQ185929.1 | 205 |
|  | R:TCCAACTCCTTGACCTCTATCC3 |  |  |

**Table S2 Network Toxicology GO Enrichment Results**

| **Go Name** | **GO ID** | **GO Category** | **pvalue** |
| --- | --- | --- | --- |
| positive regulation of gene expression | GO:0010628 | BP | 9.37E-67 |
| response to xenobiotic stimulus | GO:0009410 | BP | 1.51E-51 |
| positive regulation of apoptotic process | GO:0043065 | BP | 2.36E-47 |
| negative regulation of apoptotic process | GO:0043066 | BP | 1.66E-44 |
| apoptotic process | GO:0006915 | BP | 5.21E-38 |
| response to lipopolysaccharide | GO:0032496 | BP | 8.51E-37 |
| positive regulation of transcription by RNA polymerase II | GO:0045944 | BP | 1.38E-36 |
| response to oxidative stress | GO:0006979 | BP | 7.27E-36 |
| positive regulation of DNA-templated transcription | GO:0045893 | BP | 1.19E-34 |
| regulation of cell population proliferation | GO:0042127 | BP | 1.60E-34 |
| cytosol | GO:0005829 | CC | 1.16E-41 |
| protein-containing complex | GO:0032991 | CC | 8.26E-38 |
| cytoplasm | GO:0005737 | CC | 9.91E-36 |
| extracellular space | GO:0005615 | CC | 1.14E-33 |
| extracellular region | GO:0005576 | CC | 1.27E-29 |
| cell surface | GO:0009986 | CC | 5.24E-29 |
| nucleus | GO:0005634 | CC | 2.93E-27 |
| mitochondrion | GO:0005739 | CC | 4.46E-27 |
| perinuclear region of cytoplasm | GO:0048471 | CC | 6.92E-23 |
| nucleoplasm | GO:0005654 | CC | 7.80E-19 |
| protein binding | GO:0005515 | MF | 4.18E-76 |
| identical protein binding | GO:0042802 | MF | 3.27E-60 |
| protein-containing complex binding | GO:0044877 | MF | 6.05E-45 |
| enzyme binding | GO:0019899 | MF | 1.02E-42 |
| ubiquitin protein ligase binding | GO:0031625 | MF | 6.00E-27 |
| sequence-specific DNA binding | GO:0043565 | MF | 1.58E-20 |
| protein kinase binding | GO:0019901 | MF | 1.67E-20 |
| transcription cis-regulatory region binding | GO:0000976 | MF | 5.59E-20 |
| transferase activity | GO:0016740 | MF | 7.77E-19 |
| protein homodimerization activity | GO:0042803 | MF | 8.41E-19 |

**Table S3 Network Toxicology KEGG Enrichment Results**

| **KEGG Name** | **KEGG ID** | **p-value** |
| --- | --- | --- |
| PI3K-Akt signaling pathway | KEGG:04151 | 3.11E-50 |
| Apoptosis | KEGG:04210 | 4E-46 |
| AGE-RAGE signaling pathway in diabetic complications | KEGG:04933 | 9.03E-62 |
| FoxO signaling pathway | KEGG:04068 | 4.25E-37 |
| p53 signaling pathway | KEGG:04115 | 4.31E-34 |
| Th17 cell differentiation | KEGG:04659 | 1.1E-38 |
| HIF-1 signaling pathway | KEGG:04066 | 7.79E-34 |
| MAPK signaling pathway | KEGG:04010 | 4.83E-35 |
| JAK-STAT signaling pathway | KEGG:04630 | 9.34E-28 |
| NF-kappa B signaling pathway | KEGG:04064 | 4.93E-25 |

**Table S4** **Network Pharmacology GO Enrichment Results**

| **GO Name** | **GO ID** | **Term PValue** | **Groups** |
| --- | --- | --- | --- |
| positive regulation of signaling receptor activity | GO:2000273 | 0.001943867 | [Group12, Group14] |
| promoter-specific chromatin binding | GO:1990841 | 4.20E-04 | [Group14] |
| histone decrotonylase activity | GO:0160009 | 6.64E-10 | [Group14] |
| protein decrotonylase activity | GO:0160008 | 6.64E-10 | [Group14] |
| serotonin receptor activity | GO:0099589 | 4.29E-05 | [Group11] |
| neurotransmitter receptor activity involved in regulation of postsynaptic membrane potential | GO:0099529 | 0.004035232 | [Group11] |
| ligand-activated transcription factor activity | GO:0098531 | 1.82E-05 | [Group12] |
| cysteine-type endopeptidase activity involved in execution phase of apoptosis | GO:0097200 | 1.42E-04 | [Group03] |
| cysteine-type endopeptidase activity involved in apoptotic signaling pathway | GO:0097199 | 3.97E-04 | [Group03] |
| negative regulation of protein serine/threonine kinase activity | GO:0071901 | 3.66E-05 | [Group13] |
| metalloaminopeptidase activity | GO:0070006 | 3.55E-04 | [Group10] |
| regulation of protein tyrosine kinase activity | GO:0061097 | 2.51E-04 | [Group13] |
| carboxylic ester hydrolase activity | GO:0052689 | 7.02E-06 | [Group02] |
| Hsp90 protein binding | GO:0051879 | 1.00E-04 | [Group01] |
| NF-kappaB binding | GO:0051059 | 6.48E-04 | [Group12, Group14] |
| NADP binding | GO:0050661 | 3.54E-04 | [Group00] |
| bioactive lipid receptor activity | GO:0045125 | 1.37E-08 | [Group06] |
| positive regulation of JUN kinase activity | GO:0043507 | 0.001943867 | [Group13] |
| regulation of JUN kinase activity | GO:0043506 | 4.20E-04 | [Group13] |
| negative regulation of MAP kinase activity | GO:0043407 | 0.004221318 | [Group13] |
| proteoglycan binding | GO:0043394 | 0.001319346 | [Group05] |
| histone deacetylase binding | GO:0042826 | 6.24E-05 | [Group12, Group14] |
| acetylcholine binding | GO:0042166 | 2.13E-05 | [Group11] |
| sphingosine-1-phosphate receptor activity | GO:0038036 | 4.58E-08 | [Group06] |
| protein lysine deacetylase activity | GO:0033558 | 1.27E-11 | [Group14] |
| monocarboxylic acid binding | GO:0033293 | 2.02E-04 | [Group04] |
| deacetylase activity | GO:0019213 | 1.33E-13 | [Group14] |
| hydro-lyase activity | GO:0016836 | 1.14E-10 | [Group09] |
| carbon-oxygen lyase activity | GO:0016835 | 7.95E-10 | [Group09] |
| hydrolase activity, acting on carbon-nitrogen (but not peptide) bonds, in linear amides | GO:0016811 | 1.18E-09 | [Group14] |
| hydrolase activity, acting on carbon-nitrogen (but not peptide) bonds | GO:0016810 | 1.41E-08 | [Group14] |
| acetylcholine receptor activity | GO:0015464 | 4.76E-05 | [Group11] |
| coreceptor activity | GO:0015026 | 0.002314529 | [Group08] |
| dipeptidyl-peptidase activity | GO:0008239 | 6.39E-07 | [Group10] |
| exopeptidase activity | GO:0008238 | 2.76E-10 | [Group10] |
| metalloexopeptidase activity | GO:0008235 | 2.07E-06 | [Group10] |
| G protein-coupled amine receptor activity | GO:0008227 | 1.85E-04 | [Group11] |
| negative regulation of protein kinase activity | GO:0006469 | 2.03E-06 | [Group13] |
| collagen binding | GO:0005518 | 0.006132025 | [Group05] |
| fatty acid binding | GO:0005504 | 0.003341404 | [Group04] |
| G protein-coupled serotonin receptor activity | GO:0004993 | 4.29E-05 | [Group11] |
| prostaglandin receptor activity | GO:0004955 | 1.55E-05 | [Group07] |
| prostanoid receptor activity | GO:0004954 | 2.13E-07 | [Group07] |
| icosanoid receptor activity | GO:0004953 | 9.47E-09 | [Group07] |
| nuclear receptor activity | GO:0004879 | 1.82E-05 | [Group12] |
| non-membrane spanning protein tyrosine phosphatase activity | GO:0004726 | 3.66E-05 | [Group13] |
| protein tyrosine phosphatase activity | GO:0004725 | 2.97E-04 | [Group13] |
| JUN kinase activity | GO:0004705 | 4.96E-04 | [Group13] |
| histone deacetylase activity | GO:0004407 | 7.79E-12 | [Group14] |
| carboxypeptidase activity | GO:0004180 | 0.002314529 | [Group10] |
| aminopeptidase activity | GO:0004177 | 1.26E-09 | [Group10] |
| carbonate dehydratase activity | GO:0004089 | 4.61E-15 | [Group09] |
| p53 binding | GO:0002039 | 0.006132025 | [Group14] |
| fibronectin binding | GO:0001968 | 5.38E-04 | [Group08] |
| virus receptor activity | GO:0001618 | 6.55E-05 | [Group08] |
| amyloid-beta binding | GO:0001540 | 1.19E-04 | [Group11] |
| transcription coactivator binding | GO:0001223 | 0.001943867 | [Group12] |
| transcription corepressor binding | GO:0001222 | 9.18E-05 | [Group12, Group14] |
| transcription coregulator binding | GO:0001221 | 4.01E-05 | [Group12, Group14] |

**Table S5 Network Pharmacology KEGG Enrichment Results**

| **KEGG Name** | **KEGG ID** | **Term PValue** | **Groups** |
| --- | --- | --- | --- |
| Dilated cardiomyopathy | KEGG:05414 | 0.018524 | [Group13] |
| Arrhythmogenic right ventricular cardiomyopathy | KEGG:05412 | 0.008772 | [Group13] |
| Hypertrophic cardiomyopathy | KEGG:05410 | 0.002552 | [Group13] |
| Small cell lung cancer | KEGG:05222 | 0.002809 | [Group13] |
| Chemical carcinogenesis | KEGG:05204 | 0.036263 | [Group10] |
| Legionellosis | KEGG:05134 | 0.022153 | [Group13] |
| Nicotine addiction | KEGG:05033 | 0.008502 | [Group11] |
| Amphetamine addiction | KEGG:05031 | 0.005972 | [Group12] |
| Regulation of lipolysis in adipocytes | KEGG:04923 | 2.94E-04 | [Group09] |
| Thyroid hormone signaling pathway | KEGG:04919 | 2.75E-04 | [Group08] |
| Cholinergic synapse | KEGG:04725 | 0.006749 | [Group11] |
| Renin-angiotensin system | KEGG:04614 | 3.40E-06 | [Group07] |
| Neutrophil extracellular trap formation | KEGG:04613 | 2.89E-05 | [Group06] |
| Platelet activation | KEGG:04611 | 0.001933 | [Group05] |
| ECM-receptor interaction | KEGG:04512 | 0.013853 | [Group13] |
| VEGF signaling pathway | KEGG:04370 | 0.024247 | [Group13] |
| Notch signaling pathway | KEGG:04330 | 0.024247 | [Group12] |
| Longevity regulating pathway | KEGG:04213 | 0.027581 | [Group12] |
| p53 signaling pathway | KEGG:04115 | 0.041784 | [Group13] |
| Neuroactive ligand-receptor interaction | KEGG:04080 | 2.92E-08 | [Group04] |
| Sphingolipid signaling pathway | KEGG:04071 | 0.008361 | [Group03] |
| PPAR signaling pathway | KEGG:03320 | 0.001134 | [Group02] |
| Nitrogen metabolism | KEGG:00910 | 2.52E-12 | [Group01] |
| Arachidonic acid metabolism | KEGG:00590 | 0.003842 | [Group00] |
